# Bird-window collisions: Mitigation efficacy and risk factors across two years

**DOI:** 10.7717/peerj.11867

**Published:** 2021-07-28

**Authors:** Barbara B. Brown, Sabrina Santos, Natalia Ocampo-Peñuela

**Affiliations:** 1 Family & Consumer Studies Department, University of Utah, Salt Lake City, UT, United States of America; 2 Environmental and Sustainability Studies, University of Utah, Salt Lake City, UT, United States of America; 3 Environmental Studies, University of California, Santa Cruz, Santa Cruz, CA, United States of America

**Keywords:** Bird, Window collision/strike, Prevention, Reflective windows, Bird-friendly window, Collision deterrent film, Fritted window, UV patterned window, Cedar Waxwing, Season

## Abstract

**Background:**

Research on bird-window collision mitigation is needed to prevent up to a billion bird fatalities yearly in the U.S. At the University of Utah campus (Salt Lake City, Utah, USA), past research documented collisions, especially for Cedar Waxwings (*Bombycilla cedrorum*) drawn to fruiting ornamental pears in winter. Mirrored windows, which have a metallic coating that turns window exteriors into mirrors, had frequent collisions, which were mitigated when Feather Friendly®bird deterrent markers were applied. Bird-friendly windows–ORNILUX®ultraviolet (UV) and fritted windows–also reduced collisions when data were collected across fall and winter. Extending this prior research, we evaluated additional mitigation and tested the replicability of effects for pear trees, mirrored windows, and bird-friendly windows across two years.

**Methods:**

Using published data from eight buildings monitored for collisions in year 1 (Fall and Winter, 2019–2020), we added another year of monitoring, Fall and Winter, 2020–2021. Between years, Feather Friendly®mitigation markers were added to collision-prone areas of two buildings, including both mirrored and transparent windows.

**Results:**

The two buildings that received new Feather Friendly®mitigation had significantly fewer collisions post-mitigation. Control areas also had nonsignificant decline in collisions. The interaction of area (mitigation vs. control) by time (year 1 vs. 2) was significant, based on generalized estimating equations (GEE). The total yearly collisions across all eight buildings declined from 39 to 23. A second GEE analysis of all 8 buildings showed that mirrored windows, pear trees, and bird-friendly windows were each significant when analyzed separately. The best-fit model showed more collisions for mirrored windows and fewer collisions for bird-friendly windows. We found pear tree proximity to be related to more collisions in winter than fall. In addition, pear trees showed reduced collisions from year 1 to 2, consistent with new mitigation for two of three buildings near pear trees.

**Discussion:**

Feather Friendly^®^ markers can mitigate collisions with transparent windows, not only mirrored windows, compared to unmitigated areas over 2 years. Results also underscore the dangers of pear tree proximity and mirrored windows and the efficacy of bird-friendly windows. Thus, bird collisions can be prevented by window mitigation, permanent bird-friendly windows, and landscape designs that avoid creating ecological traps.

## Introduction

Threats to birds from windows are becoming well-known, with a systematic review estimating up to one billion birds per year killed in the U.S. in window collisions (Loss et al., 2014). Birds fly into windows because they do not see transparent windows as solid surfaces or they see reflected images of sky or habitat as places to fly through (Klem, 1989). Past research documents how new window materials or applications of mitigation material to existing windows can reduce these collisions (Klem, 2009; Klem & Saenger, 2013; Ocampo-Peñuela, Peñuela Recio & Á, 2016a; Sheppard, 2019; Swaddle et al., 2020). Much of the research on bird-friendly window treatments is drawn from special tunnel tests that offer controlled flight conditions in the presence of specially mitigated windows. These essential tunnel tests should be complemented with additional research on existing buildings that include pre-test and post-test measures of both experimental areas receiving mitigation and control areas that do not receive mitigation. Such studies, often characterized as quasi-experimental designs (Cook & Campbell, 1979) or before-after control-impact (BACI) designs (Underwood, 1992), can identify the range of building-related qualities, such as windows and landscaping, that could be modified to reduce bird-window collisions (Basilio, Moreno & Piratelli, 2020; Dunn, 1993; Hager et al., 2008). Therefore, it is important to study the efficacy of window treatments as well as the risk factors that exist *in situ*, in order to reduce the mortality associated with the most dangerous buildings.

The current study extends past research conducted on the University of Utah campus in Salt Lake City, Utah, USA (Brown et al., 2019). A mirrored building façade had a metallic coating that turned the window exterior into a mirror under most lighting conditions. The building with the mirrored facade was near ornamental pear trees (*Pyrus calleryana*) that drew Cedar Waxwings (*Bombycilla cedrorum*) to feed but also to collide with the mirrored facade. A bird collision deterrent film, applied to part of the mirrored façade, reduced collisions in the second winter. In addition, a correlational follow-up of eight buildings during the second winter identified nearby pear trees and mirrored windows as the best predictors of collisions. Although two buildings had permanent bird-friendly windows, fabricated with frits or ultraviolet-reflective patterned windows, overall collisions in winter were so low that the permanent bird-friendly windows did not have fewer collisions than the other six buildings (Brown, Hunter & Santos, 2020). However, when data collection was expanded the next year to include both fall and winter seasons, the numbers of collisions overall naturally increased and the two buildings with permanent bird-friendly windows exhibited fewer collisions than other buildings, given the greater variability and total numbers of collisions introduced by extending observations to fall. The current study extends these past two analyses by evaluating additional mitigation for transparent windows, not just mirrored windows. In addition, a new fall and winter season of data collection across all eight buildings allows us to test the stability of risk factors across two years in order to establish greater generalizability of findings.

### Untreated windows

Unless specially treated, many windows currently installed in buildings pose dangers to birds because transparent windows are not perceived as a solid surface or reflections from clear or tinted windows are seen as more habitat. (Klem, 2009). One way to understand bird avoidance of window surfaces is from research in specially designed tunnels, where a released bird is observed to swerve toward one of two window treatments (Klem, 2009; Rössler, Nemeth & Bruckner, 2015; Sheppard, 2019; Swaddle et al., 2020). Alternatively, research assesses how many birds fly into different types of windows erected in fields near woods (Klem, 1989; Klem, 1990; Klem, 2009). Both the controlled tunnel tests and the more open field tests show that birds will frequently fly into windows unless they have been mitigated with visible markers affixed to the external surface in a 5 x 10 cm (2 x 4 inch) pattern or when windows are specially fabricated with materials that are visible to birds. Outside of testing situations, multiple studies have confirmed that buildings with unprotected glass are vulnerable to collisions, especially buildings with greater amounts of glass (Basilio, Moreno & Piratelli, 2020; Borden et al., 2010; Cusa, Jackson & Mesure, 2015; Elmore et al., 2020; Hager et al., 2013; Hiemstra, Dlabola & O’Brien, 2020; Keyes & Sexton, 2014; Klem et al., 2009; Ocampo-Peñuela et al., 2016b; Riding, O’Connell & Loss, 2019; Schneider et al., 2018).

Buildings with mirrored glass, which is reflective under a wide range of lighting conditions, appear to be especially dangerous to birds, according to numerous anecdotal observations in past studies (Evans Ogden, 2002; O’Connell, 2001; Sheppard & Phillips, 2015). The systematic test of mitigation efficacy for the mirrored façade at the University of Utah used Feather Friendly^®^ Symmetry pattern bird collision deterrent. This deterrent is applied as a film onto the window exterior, then the backing is removed, leaving small (six mm.) white dots, spaced 5.08 cm apart. These markers are designed to make the window visible to birds as a solid surface. The dots can be seen by humans, although they do not block much total area of the view. By affixing the dots to 36 m^2^ of a 199 m^2^ mirrored surface, collisions were reduced by 71% on the mitigated surface and unchanged on the remaining untreated mirrored or traditional transparent glass surfaces of the building (Brown et al., 2019). Specifically, from winter 1 (November 9, 2017 to January 2, 2018) to winter 2 (November 15, 2019 to January 12, 2019) the mitigation area had collisions decline from seven pre-mitigation to two post-mitigation, a 71% reduction, while remaining at 8 collisions on unprotected windows in both years. As one would expect when a mitigation is effective, the treatment by year interaction effect was significant (*p* = 0.03) in a generalized estimating equation test, supporting the efficacy of the Feather Friendly^®^ treatment.

### Bird-friendly windows

A growing number of windows are manufactured to include features that may be visible to birds, including fritted windows and ultraviolet-reflective patterned windows. Fritted windows have small ceramic frits fused into the glass and are employed primarily to reduce heat and light penetration (Wilson & Elstner, 2018). However, these windows often deter bird collisions as well. In outdoor cage tests, birds faced with a choice of fritted or clear windows avoided flying toward the fritted windows in 89% of 70 trials (Klem, 2009). These frits were 0.32 cm in diameter and 0.32 cm apart; thus, they may be less acceptable to humans who want unobstructed window views.

Windows manufactured with patterns that reflect ultraviolet light will reflect short wavelengths of light that are visible to many birds, but are less detectible by humans. For example, the ORNILUX^®^ Mikado pattern will, upon close examination by a human, appear to have patterns loosely resembling pick-up sticks embedded in the window, but without obstructing the view. Birds may see these patterns more readily than humans, though more research about bird vision across species and viewing conditions is needed. The Mikado pattern was inspired by ultraviolet-reflective strands of webs created by orb weaver spiders that may warn off approaching birds (Postel, 2016). In outdoor field tests ORNILUX Mikado pattern deterred collisions 55% of the time when the window covered a darkened interior, which was comparable to the 58% to 66% deterrence when windows were tested in tunnels (Klem & Saenger, 2013). However, bird species differ in their ability to see ultraviolet reflections (Håstad & Ödeen, 2014) and these reflections may be less visible on cloudy days (Sheppard, 2019). Together the fritted and ORNILUX^®^ windows were associated with fewer collisions at the University of Utah when data were collected for both fall and winter seasons (Brown, Hunter & Santos, 2020). Given few tests of these products in existing buildings, replications are warranted.

### Trees and collisions

In addition to considering the dangers posed by building windows, the landscape surrounding each building may attract or repel birds, altering their likelihood of collisions. Past research has found that trees or shrubs close to buildings may attract birds and reflections of trees or shrubs in windows or mirrored surfaces can mask the solid hazard of glass, increasing collisions (Borden et al., 2010; Gelb & Delacretaz, 2006; Klem et al., 2009; Kummer, Bayne & Machtans, 2016b; Loss et al., 2019), although others find no effect (Hager et al., 2017). Few studies have examined whether particular trees are associated with more collisions, although some studies have shown that taller trees near buildings relate to more collisions (Klem et al., 2009; Kummer, Bayne & Machtans, 2016b). Another study found that trees with fruits or berries attract a greater variety of native birds, but did not examine collisions (Belaire, Whelan & Minor, 2014). Fruit trees became a focus at the current study site because of past years of sightings of Cedar Waxwings feeding on pear trees in winter (Brown, Hunter & Santos, 2020; Brown et al., 2019). Waxwings are especially dependent on fruit as a food source in winter (Witmer, 1996; Witmer, Mountjoy & Elliot, 2014). Thus, while some studies highlight the dangers of any surrounding trees or shrubs, the current study focuses on the dangers of pear trees near buildings with mirrored or transparent windows.

In sum, this study addressed three research questions. First, we tested whether the application of Feather Friendly^®^ bird deterrent markers reduced bird collisions across two years, compared with control areas. Second, we assessed whether nearby pear trees and mirrored windows related to greater collisions while bird-friendly windows (fritted and ORNILUX) related to fewer collisions. Third, we examined seasonal differences in collisions, attempting to replicate the associations found between pear trees and winter collisions, especially for Cedar Waxwings, compared with fall.

## Methods

### Study site and buildings monitored

The University of Utah campus is designated as a state arboretum and is landscaped with a combination of native and introduced trees. Native species include blue spruce (*Picea pungens*), white fir (*Abies concolor*), and Gambel oak (*Quercus gambellii*). Introduced species include English hawthorn **(***Crataegus laevigata*) and European horsechestnut (*Aesculus hippocastanum*). Fruiting trees such as ornamental Callery pear trees (*Pyrus calleryana*) and flowering crabapples (multiple *Malus* species) are common on campus. The campus is on the northeast edge of Salt Lake City, between the city and the foothills of the Wasatch mountains (Level III US Environmental Protection Agency Ecoregion 19, Environmental Protection Agency, 2018).

The eight buildings that had been observed in fall 2019 to winter 2020 (Brown, Hunter & Santos, 2020) were monitored again, using the past data as the year 1 baseline data for the current study (map available as Fig. 2 in Brown, Hunter & Santos, 2020). These buildings were chosen initially in consultation with grounds crews, ornithologists, and bird watchers on campus. Table 1 describes the characteristics, glass area and treatment of every building sampled. Buildings were considered to be near pear trees when within 4.9 m.

**Table 1:** Building characteristics and total collisions by building, season, and year. Building 1 also had 119 m^2^ of mirrored windows. Similar dates were used for data collection in Year 1 (Fall: September 12–October 27, 2019; Winter: October 29, 2019–January 24, 2020) and Year 2 (Fall: September 11–October 28, 2020; Winter: October 30, 2020–January 27, 2021). Data were collected at the University of Utah, Salt Lake City Utah, USA. Monitoring included all four sides (for buildings 2, 3, 4, 5, and 7) or certain sides (for 1, 6, and 8) of each building.

					**Collisions**						
**Building**	**Risk/protective features (0 = absent, 1 = present)**			**Window area**, m^**2**^	**Year 1**			**Year 2**			**Year 2 - 1**
**Number & initials**	**Pear trees**	**Mirrored windows**	**Bird-friendly glass**		**Fall**	**Winter**	**Total**	**Fall**	**Winter**	**Total**	**Totals**
1. AEB	1	1	0	144	0	14	14	0	5	5	−9
2. JTB	1	0	0	431	1	10	11	0	2	2	−9
3. CSC	0	0	0	660	0	1	1	0	2	2	1
4. LCB	1	0	0	788	1	0	1	1	1	2	1
5. MEB	0	0	0	3,342	4	1	5	1	3	4	−1
6. WEB	0	0	0	1,219	4	1	5	3	1	4	−1
7. Law	0	0	1	1,860	2	0	2	1	1	2	0
8. GC	0	0	1	1,002	0	0	0	1	1	2	2
Totals					12	27	39	7	16	23	16

Between years 1 and 2, a windstorm with 129 –161 kph winds occurred on campus. Many trees were toppled or damaged, with substantial damage to a green area between study buildings 1 through 4 (Mitchell, 2020). Two of our study buildings (buildings 1 and 2, Table 1) had one tree each removed from the building perimeter where our monitors looked for birds. Although none of the pear trees that typically provide winter food to Cedar Waxwings were toppled, some leaves and small branches were damaged. Later observations suggested that the pears did not remain on the trees as long as in past years. It is unclear whether this was due to the storm, drought conditions (NOAA, 2021), or increased foraging by invasive fox squirrels (*Sciurus niger*) (Welch, 2018). Furthermore, other birds, including European Starlings (*Sturnus vulgaris)*, American Robins (*Turdus migratorius*), and House Finches (*Haemorhous mexicanus)* also fed upon the pears and could have reduced fruit availability.

### Intervention

The mirrored façade on building 1 had been partially treated during the 2019 round of mitigation (Brown et al., 2019); the current round of mitigation covered the remaining mirrored surface on the second and third floors and also covered three historical transparent windows that faced a line of pear trees. Building 2 had never received mitigation and its long north side faced the line of pear trees that attracted Cedar Waxwings and other fruit-eaters. Building 1 received 47.71 m^2^ of new mitigation (42.38 m^2^ on mirrored, 5.33 m^2^ on transparent windows); building 2 received 59.86 m^2^ of new mitigation on transparent windows (57.38 m^2^ on north face, 2.48 m^2^ on west face). Installation was completed in August, 2020. These applications were targeted toward the deadliest accessible windows on the buildings and did not include all glass surfaces. Some areas that had bird collisions in the past could not be easily accessed due to railings and other structures beneath the windows and the need for more specialized lifts. Costs of labor, materials and lift rental were approximately $29,000 and financed by a campus sustainability fund; these details are provided following recommendations that this information be shared to benefit other collision prevention groups (Brown et al., 2019).

### Collision monitoring

Collisions were defined as any stunned birds, fatalities, or feather piles (>12 feathers within 0.09 m^2^) found under windows and extending 3 m from the designated building sides. Three trained monitors verified their building observations by taking time-stamped cellphone photos of each building they observed. We uploaded photos to a shared drive. We double-bagged carcasses or feather piles and made them available to an ornithology lab on campus that had salvage licenses (United States Fish and Wildlife Service permit # MB836059-0, and Utah Division of Wildlife Resources Certificate of Registration # 5COLL3669) for research and disposal (Hager & Cosentino, 2014). Study procedures received permission for data collection from the University of Utah institutional review board (IRB_00117279). We photographed all carcasses and stunned birds, where possible, and uploaded the images to an iNaturalist project (see https://www.inaturalist.org/projects/university-of-utah-bird-window-collision-project). Our monitoring rules meant that some observations logged into the iNaturalist repository were incidental and thus not included in our analyses, such as a stunned Cedar Waxwing observed under unprotected windows of building 1 on a non-observation day.

#### Data collection dates and times

Year 1 fall monitoring occurred September 12 to October 27, 2019 and winter monitoring occurred from October 29, 2019 to January 24, 2020, with 224 of those observations involving paired observers monitoring the same buildings (Brown, Hunter & Santos, 2020). In year 2 we conducted monitoring during a similar time frame, with fall monitoring from September 11 to October 28, 2020 and winter from October 30, 2020 to January 27, 2021, with 203 paired observations. We typically monitored three times a week with at least one day between observations, given past research that suggested this interval should allow us to detect most carcasses, based on studies of carcass removal by predators and scavengers (Hager, Cosentino & McKay, 2012; Hager & Craig, 2014; Kummer et al., 2016a; Riding & Loss, 2018). October 7, 2020, was an exception, when a security perimeter (for the US Vice Presidential debate) blocked us from observing four of the eight buildings. For that week, monitoring was made up by taking three weekly observations, but only one day apart for two of the observations. Observation times during the day varied according to observer availability.

#### Interrater reliability data

A subset of the observations included two raters to assess interrater reliability. This involved two observers, travelling in opposite directions, around the same building at a similar time. Observers in year 1 were instructed that if they found a bird during a reliability check they should take a quick picture of it immediately, so as not to delay completion of the route and thereby forewarn the other rater of the presence of a carcass. In year 2, observers used the same procedure if conducting simultaneous observations. If observers needed to be socially distanced in year 2 to prevent spread of Covid-19, the observers would compare collision sightings after both observers had finished the target buildings. For each observation, photographs included close-up and more distant shots that identified the section of the building for each collision.

### Data analysis procedures

We assessed interrater reliability with the intraclass correlation coefficient (ICC); this statistic, ranging from 0 to 1.0, shows perfect agreement at 1.0 (Shrout & Fleiss, 1979). The ICC scores are interpreted as evidence of good reliability if the 95% confidence intervals (CI) contain values between 0.75 and 0.90 and excellent reliability if values are above 0.90 (Koo & Li, 2016). We selected the value suitable for reliance on a single rater’s score rather than for an average score across observers (Koo & Li, 2016) and used the one-way random effects approach for multiple observers. All analyses used IBM SPSS Statistics 26 software (Version 26.0; IBM, Armonk, NY, USA)

To assess whether installing Feather Friendly^®^ bird-deterrent film was effective, we used generalized estimating equations (GEE), a technique appropriate for count data and repeated measures (Ma, Mazumdar & Memtsoudis, 2012; Norusis, 2008), which is recommended when one’s model needs to take into account data clustered within buildings but without the need for random effects of a Generalized Linear Mixed Model (GLMM) model (West, Welch & Galecki, 2015). Instead, GEE is based on a marginal model, where the goal is to estimate mean collisions by the risk factors across buildings (Carrière & Bouyer, 2002). We used a Poisson distribution with a log-link function and an auto-regressive correlation structure across repeated measures. Bird strikes, the outcome or response variable, are summed across each week of observation, to replicate the procedure from Brown et al. (2019). The first factor in the model includes the within-subjects factor of time, with pre-mitigation observations during year 1 and post-mitigation observations during year 2. The second factor is the between-group variable of window area of the building, with control areas unchanged across both years and the mitigation areas receiving protective film between years 1 and 2. Building 1 had one collision in year 1 that could not be assigned to either treatment or control areas, given that the feathers and bits of carcass had been smeared across both areas, likely by a predator. This case was dropped from the analysis of treatment effects but retained in descriptive and other analyses that did not require designation of treatment and control areas. If mitigation reduces collisions, we would expect to see a reduction of collisions across years for the treatment area compared with control area. This treatment effect would be represented by a significant (*p* = 0.05) area by year interaction effect, with simple effects follow-up tests using Wald *χ*^2^ tests of significance.

To assess whether collisions are related to the risk factors of proximity to pear trees and presence of a mirrored window, and to the protective factor of the presence of bird-friendly permanent windows (fritted or ORNILUX^®^ UV windows), a GEE analysis was used for all eight buildings across two years. Again, a Poisson distribution with a log-link function was used. We used the simple independent correlation structure across repeated measures after finding that an auto-regressive correlation structure yielded similar model fit. Our design treated the eight buildings as a repeated factor and nested the temporal characteristics of observation day and year as within-subject factors within each building. To follow past procedure from Brown, Hunter & Santos (2020), we tested the three environmental features—pear trees, mirrored windows, and bird-friendly windows—together and separately, and also defined the best fit model of all possible 1-, 2-, and 3-variable combinations. The fit statistic was the QICC, the quasi-likelihood under independence model criterion, in which models with lower scores indicate better fit (Norusis, 2008).

To replicate whether pear tree proximity was especially hazardous for birds in winter, we tested a pear tree by season interaction effect. If more collisions occur with winter pear tree proximity, the interaction should be significant. In this data set, unlike prior data sets, we have two full years of assessment of the risk factors. Two years of data allow us to test for significant interactions by year, to assess the stability of risk factors across years. For interactions with season or year we present only significant interaction effects.

## Results

### Reliability and descriptive information

Reliability checks showed that observers had good to excellent agreement, with ICCs of 0.92 in year 1 (95% CI [0.89–0.94]) and 0.92 in year 2 (95% CI [0.90–0.94]).

Fewer collisions across all 8 buildings were noted in year 2 (*n* = 23) than in year 1 (*n* = 39) (Table 1). Six of the eight buildings had fairly stable collision numbers, which varied only +/−2 collisions. However, the two treated buildings had the greatest number of collisions during year 1 and also had the largest decline in strikes, with declines of 9 collisions for each of buildings 1 and 2.

Of the 62 total collisions, 30 (48.39%) involved Cedar Waxwings in winter (Table 2). Dark-eyed Juncos (*Junco hyemalis)* had 8 collisions across fall and winter, with the remaining species represented by 3 or fewer collisions. In total, 18 identifiable species were involved in collisions.

**Table 2:** Number of window collisions per species by building and fall (F) and winter (W) season.

		**Building number**								
**Common name**	**Scientific name**	**1**	**2**	**3**	**4**	**5**	**6**	**7**	**8**	**Totals**
Cedar Waxwing	*Bombycilla cedrorum*	14-W1 5-W2	9-W1 1-W2		1-W2					30
Dark-eyed Junco	*Junco hyemalis*			2-W2	1-F2	2-W2	1-F1 1-F2 1-W2			8
Lincoln’s Sparrow	*Melospiza lincolnii*					1-F2	1-F1	1-F1		3
Orange-crowned warbler	*Leiothlypis celata*					1-F1		1-F2		2
Yellow-rumped Warbler	*Setophaga coronata*						1-F1, 1-W1			2
Mourning Dove	*Zenaida macroura*							1-F1	1-F2	2
Red-naped Sapsucker	*Sphyrapicus nuchalis*		1-F1							1
Black-capped chickadee	*Poecile atricapilla*					1-W1				1
Townsend’s Solitaire	*Myadestes townsendi*					1-F1				1
American Robin	*Turdus migratorius*		1-W1							1
House Finch	*Haemorhous mexicanus*				1-F1					1
Brewer’s Sparrow	*Spizella breweri*					1-F1				1
Lazuli Bunting	*Passerina amoena*						1-F1			1
Rock Pigeon	*Columba livia*		1-W2							1
Brown Creeper	*Certhia americana*					1-W2				1
MacGillivray’s Warbler	*Geothlypis tolmiei*						1-F2			1
Spotted Towhee	*Pipilo maculatus*						1-F2			1
Ruby-crowned Kinglet	*Regulus calendula*							1-W2		1
Unknown bird				1-W1		1-F1			1-W2	3
Totals		19	13	3	3	9	9	4	2	62

**Notes.**

W winter F fall season 1 year 1 2 year 2

### Feather Friendly^®^ mitigation evaluation

**Figure 1:**
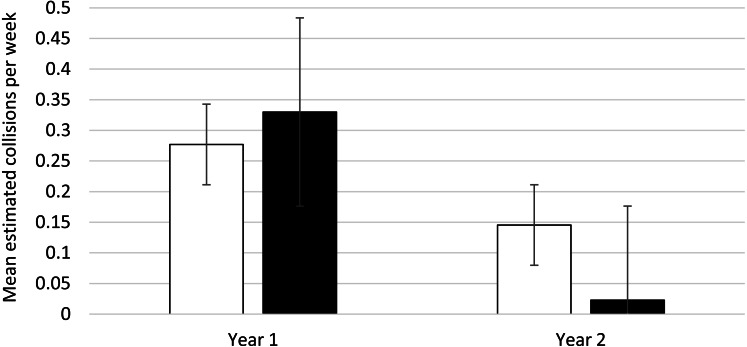
Bird collisions per week, pre- and post-mitigation: estimated marginal means (and error bars) from generalized estimating equations. Control area: White bar; Treated area: Black bar. Estimated means with error bars are shown. The treated area had mitigation applied between year 1 and 2; the control area did not. The data were gathered fall and winter from two buildings at the University of Utah, Salt Lake City Utah, USA. Year 1 included September 12, 2019–January 24, 2020. Year 2 included September 11, 2020–January 27, 2021.

Buildings 1 and 2, which received Feather Friendly^®^ mitigation treatment between years 1 and 2, had 24 collisions that could be assigned to either mitigation or control areas in year 1 and 7 in year 2. Consistent with the overall reduction of collisions in year 2, collisions for both treatment and control areas decreased in year 2. Control areas collisions declined from 11 to 6 (a 45.45% reduction), and treatment areas declined from 13 to 1 (a 92.31% reduction). The weekly collision averages across years, based on the expected marginal mean from the GEE analysis, are indicated on Fig. 1. The interaction of area by time was significant, indicating different amounts of change in collisions over time for treatment and control areas (Wald *χ*^2^ = 14.99, *d.f.* = 3, *p* = 0.002). The follow-up simple effects tests show the decrease in collisions was significant for the experimental area (Wald *χ*^2^ = 8.09, *d.f.* = 1, *p* = 0.004) but not for the control area (Wald *χ*^2^ = 1.24, *d.f.* = 1, *p* = 0.27). Thus, the results support the effectiveness of the mitigation.

### Risk factors across years 1 and 2

Table 3 summarizes the effects of the three environmental predictors—pear trees, mirrored windows, and permanent bird-friendly windows—on collisions across the two years. These analyses contain the three predictors together and then separately, along with the best fitting model among all possible combinations of the three predictors. When all three predictors are in Model 1, mirrored windows explained a significant amount of unique variance (*p* = 0.01), increasing the likelihood of a collision by 2.38. Given correlations among the predictors (phi coefficients range from −0.45 to 0.49, *p* = .01), Models 2 through 4 evaluate each predictor alone. In these single-variable models, each predictor is significant in the expected direction. Pear trees and mirrored windows increase odds of collisions (Exp(B) or odds ratio = 2.16 and 3.09, respectively) and bird-friendly windows reduce odds (Exp(B) = 0.32). A comparison of QICC fit statistics across all single, double, and triple predictors showed that the best fit Model 5 included mirrored windows and bird-friendly windows (QICC = 388.53 compared with 391.57 to 403.49 for other models). Again, mirrored windows increase odds (Exp(B) = 2.57) and bird-friendly windows reduce odds (Exp(B) = 0.41).

**Table 3:** Bird-window collisions per day predicted by building risk and protective features: Generalized estimating equation results.

					95% Wald C.I.	
	B	S.E.	*p*	Exp(B)	Lower	Upper
Model 1: All three predictors						
Intercept	−2.84	0.22	0.01	0.06	0.04	0.09
Pear trees	0.13	0.33	0.69	1.14	0.60	2.19
Mirrored windows	0.87	0.34	0.01	2.38	1.22	4.62
Bird-friendly glass	−0.85	0.46	0.07	0.43	0.17	1.06
Model 2: Pear trees only						
Intercept	−3.10	0.19	0.01	0.05	0.03	0.07
Pear trees	0.77	0.26	0.01	2.16	1.31	3.57
Model 3: Mirrored windows						
Intercept	−2.97	0.15	0.01	0.05	0.04	0.07
Mirrored windows	1.13	0.28	0.01	3.09	1.80	5.31
Model 4: Bird-friendly glass						
Intercept	−2.55	0.13	0.01	0.08	0.06	0.10
Bird-friendly glass	−1.14	0.43	0.01	0.32	0.14	0.75
Model 5: Mirrored + bird-friendly windows, best fit						
Intercept	−2.79	0.16	0.01	0.06	0.05	0.09
Mirrored windows	0.94	0.28	0.01	2.57	1.48	4.46
Bird-friendly windows	−0.90	0.44	0.04	0.41	0.17	0.96

**Notes.**

B B coefficient S.E. Standard error of B Exp(B) odds ratio CI confidence interval

Data were collected in fall and winter, 2019–2020 and 2020–2021 at the University of Utah, Salt Lake City, Utah, USA.

To ascertain whether buildings near pear trees posed special dangers to birds that feed on fruit in winter, such as Cedar Waxwings, we computed interactions with the three building risk factors and the two temporal variables of season and year. Table 4 shows that, once again, the presence of pear trees is especially predictive of collisions in winter, when pear trees provide an important food source for waxwings and other fruit eaters. Specifically, the expected pear tree by season interaction effect was significant (Wald *χ*^2^ = 12.00, *d.f.* = 3, *p* = 0.01). Significant simple effects follow-up tests reflect the greater number of winter collisions than fall collisions for buildings near pear trees (Wald *χ*^2^ = 8.12, *d.f.* = 1, *p* = 0.01); adjusted means show 0.08 collisions/observation day in winter compared to 0.01 in fall. Conversely, the five buildings that were not close to pear trees had more fall than winter collisions (Wald *χ*^2^ = 4.27, *d.f.* = 1, *p* = 0.04); adjusted means show 0.04 collisions/day in winter compared to 0.10 in fall.

**Table 4:** Interactive effects of pear tree proximity, season, and year on bird-window collisions: Generalized estimating equation results.

					95% Wald C.I.	
	B	S.E.	*p*	Exp(B)	Lower	Upper
Intercept	−2.35	0.34	0.01	0.10	0.05	0.18
Pear trees	−1.37	0.70	0.05	0.26	0.06	1.01
Mirrored windows	0.86	0.34	0.01	2.38	1.22	4.62
Bird-friendly glass	−0.85	0.46	0.07	0.43	0.17	1.06
Season (winter =1)	−0.99	0.39	0.01	0.37	0.17	0.80
Year (2020-2021 = 1)	0.07	0.39	0.85	1.08	0.51	2.29
Pear trees * Season	2.74	0.72	0.01	15.52	3.79	63.59
Pear trees * Year	−1.13	0.55	0.04	0.32	0.11	0.94

**Notes.**

B B coefficient S.E. Standard error of B Exp(B) odds ratio CI confidence interval

Data were collected in fall and winter 2019-2020 and 2020-2021 at the University of Utah, Salt Lake City, Utah, USA.

The only significant difference in effects of risk factors by year involved the pear tree by year interaction effect (Wald *χ*^2^ = 8.61, *d.f.* = 3, *p* = 0.04). The effect reflects what would be expected from additional mitigation of two of the three buildings near pear trees between years 1 and 2. These buildings had fewer collisions in the second year, after mitigation (Wald *χ*^2^ = 4.21, *d.f.* = 1, *p* = 0.04); adjusted means decreased from 0.06 to 0.02 across the two years.

## Discussion

### Mitigation efficacy

The addition of Feather Friendly^®^ treatment in high-collision areas of two buildings showed a significant reduction of bird collisions, supporting its efficacy. In past research, mitigation was effective for mirrored windows (Brown et al., 2019) and in the current study we confirmed its mitigation efficacy also on transparent glass. Our study contributes to important tests of collision prevention mechanisms in “real world” settings, which complement the experiments done on wind tunnels. This is imperative because what works in tunnel tests may not be as effective under less controlled settings in the built environment. Each collision may reflect influences from the building façade, the building itself, the surrounding site, and even regional or continental factors. In addition, changing seasons, lighting, and weather conditions also impact collisions. Hager et al. (2013) assert that each building has its own “mortality signature” based on these varied influences . For mitigation measures to be found effective across many field situations, researchers need to provide more quasi-experimental research findings like these to support evidence-based mitigation.

Past research has provided few examples of studies that were able to include the key features of quasi-experimental design that help strengthen causal claims for Feather Friendly^®^ mitigation. For example, a University of Pennsylvania technical report showed a 100% decline in collisions for a Feather Friendly^®^ mitigated area and a 75% decline for a control area, but did not offer statistical tests or collect data across similar seasons (Durrance, 2015). Similarly, a study at Duke University reported 88% reductions in spring and 53% reductions in fall, but the mitigated glass walkaway and towers likely did not provide similar control areas, and statistical tests were not done due to a small sample size (Winton, Ocampo-Peñuela & Cagle, 2018). Another technical report did not include a full post-test, although early results (standardized monitoring decline from 35 to 2 collisions) seem very promising (De Groot, 2018). Results also are consistent with a variety of studies that use other forms of closely spaced markers on window exteriors (Ocampo-Peñuela, Peñuela Recio & Á, 2016a; Ribeiro & Piratelli, 2020). In challenging field situations, it can be difficult to find comparable control and mitigation areas, fund the purchase and installation of mitigation material, conduct same-season pre- and post-test data collection, and provide sufficient samples for statistical testing. Though few in number, all these studies suggested that Feather Friendly^®^ mitigation was effective, with one past study providing a strong quasi-experimental design (Brown et al., 2019). The current study also supports a strong claim of efficacy of Feather Friendly^®^ mitigation markers because the key aspects of quasi-experimental research were in place: pre- and post-test data, similar seasons, control and treatment areas, and statistical analyses that can support causal claims. We encourage researchers to adopt these key aspects of strong study designs when testing products that have shown promising results from tunnel testing (Klem & Saenger, 2013; Sheppard, 2019), such as Feather Friendly^®^ or other markers (e.g., lines, squares), hanging cords (Klem & Saenger, 2013), or tempera paint (American Bird Conservancy, 2021b).

### Risk and protective factors over two years

Our test of risk and protective factors across all eight buildings largely replicated what had been found at this site in the past (Brown, Hunter & Santos, 2020). Pear trees and mirrored windows, when tested alone, were each significant predictors of collisions, while bird-friendly windows, in the form of fritted or ORNILUX UV-patterned windows, were associated with fewer collisions. The only difference between the findings of the current study and Brown, Hunter & Santos (2020) is that the best fitting model in the current study involved both mirrored windows and bird-friendly windows. In the past research, the best-fit was obtained by the two risk factors of mirrored windows and pear trees. Thus, the role of pear trees diminished as two of the three buildings near pear trees received mitigation by the Feather Friendly^®^ markers.

The seasonal vulnerability created by the fruiting pear trees that was found in the last publication (Brown, Hunter & Santos, 2020) was replicated with the new data, reflecting the fact that waxwings are dependent on fruiting trees for winter food. This dependence makes them especially vulnerable to collisions in the winter. Even though the data collection included fall, when waxwings are not yet drawn to the ornamental pears, Cedar Waxwings constituted 30 of the 62 collisions documented across the two years. In addition, a year by pear tree interaction reflected the reduction in collisions in year 2, which is consistent with the fact that the deadliest areas on the deadliest two buildings (buildings 1 and 2) near the pear trees had been mitigated.

Beyond identifying that mitigation and bird-friendly windows are useful and that pear trees and mirrored windows are harmful, we also can speculate that certain changing factors we noted in year 2 also might affect waxwing vulnerability. There was a drought in year 2 (NOAA, 2021), a damaging wind storm prior to year 2 (Mitchell, 2020), and it appeared that pear trees had less abundant and long-lasting fruit for pear trees in front of buildings 1, 2, and 4 on the northwest part of campus. Perhaps consequently, we noticed fewer waxwings feeding in the pear trees in year 2 while we collected data, although we did not document this systematically. From past and current research, during winter data collection for years 2017–2018, 2018–2019, and 2019–2020, waxwing carcasses were found from November into January. But in winter of 2020–2021, the last waxwing carcass was collected November 23, 2020, with no carcasses in December 2020 or January 2021. Meanwhile, on the southeastern edge of campus, larger than normal waxwing flocks were recorded through January by a long-time observer who logged results into the eBird app (e.g., Maguire, 2021); this alteration of feeding grounds is consistent with the nomadic tendencies of waxwings (Witmer, Mountjoy & Elliot, 2014). Maguire (2021) suggested that the pear trees, which were located close to the west side of buildings, were better protected from the windstorm from the east in September 2020. We visited the southeastern site and noted that the pear trees grew abundant and long-lasting fruit compared with the trees outside of buildings 1, 2, and 4. In addition, building 1 lost a tree in the windstorm, and for the first time in four years, 3 carcasses were found under the windows of that now-exposed corner of the building. Finally, we also speculate that the reason building 4 has consistently recorded few waxwing fatalities, despite proximity to the pear trees, is that the windows have failed. That is, water seepage in earlier years has turned the external surface of these windows visible, with a translucent, filmy appearance. Therefore, we suggest that dynamic features like a drought or a windstorm, the loss of a tree, and the failure or replacement of windows all may alter waxwing vulnerability to window collisions. In order to provide long-term protection, permanent standards for selecting or replacing windows or landscaping would help address the changing threats to waxwings and other birds.

In sum, the current research, combined with past research, suggests that it is important to consider both landscape and window factors when identifying and mounting efforts to mitigate window collisions. Both the presence of glass and vegetation have been cited as factors associated with collisions in a recent review of 53 publications (Basilio, Moreno & Piratelli, 2020). The persistent importance of pear trees that draw in frugivorous birds like Cedar Waxwings has been amply demonstrated by their overabundance in our collision numbers and by their reduction in collision numbers after mitigation. Callery pears, originally imported from China (Culley, 2017), have become an invasive species (Tallamy, 2009), yet are still popular as street trees in local suburban developments (Avolio et al., 2018). We believe humans have created an ecological trap for waxwings. Waxwings are drawn to the pears that are planted or spreading invasively; but when these trees are near windows, waxwings die from window collisions (Battin, 2004). A similar trap existed along a Texas highway when silverberry shrubs (*Elaeagnus pungens*) planted in a highway median attracted waxwings. The passing cars flushed and hit the birds, with 298 fatalities in one month (Dowler & Swanson, 1982). Few studies highlight the vulnerabilities of pairing particular trees or landscaping to particular bird species (Weldon & Haddad, 2005), although woody habitat near buildings have been linked to collisions by Swainson’s Thrush (*Catharus ustulatus*) (Elmore et al., 2020). However, given the growing presence of pear trees in the built environment, it is likely that other sites provide similar ecological traps for waxwings and perhaps other species; this supposition merits additional research.

### Limitations

The data for the study derive from a small sample of eight buildings. Likewise, the efficacy of the Feather Friendly^®^ intervention treatment was limited to a subset of windows in two buildings, although that limitation also meant that suitable control areas were available for observation on the same buildings. The number of birds in the Salt Lake City region is small compared with bird populations near coasts or major flyways. Nevertheless, some of the same risk and protective factors identified elsewhere were confirmed to operate similarly in this less-studied region. An additional constraint was that our observers were not available at the same time each day for scheduled observations, but often observed in the late morning or early afternoon. Although some researchers recommend observing birds in the early morning after migrants have collided with buildings overnight (Nichols et al., 2018) and others recommend mid- to late-afternoon (Hager & Cosentino, 2014), we have noted that many of the Cedar Waxwing collisions that dominate our study occur across the morning and afternoon. We also recognize that our observations may have missed some birds, which may have been overlooked or taken away by predators prior to observation (Klem et al., 2004; Kummer et al., 2016a; Riding & Loss, 2018). This limitation typically affects all studies that require human monitoring for bird carcasses (Hager et al., 2013) and was addressed with observer training and inter-rater reliability calculations that indicated good to excellent reliability. Finally, we share the limitation of all quasi-experimental studies that important covariates may have been omitted. Furthermore, the statistical modeling could not address, for example, building-specific changes within seasons (random slopes), which would require larger samples to accommodate Generalized Linear Mixed Models (GLMM) instead of GEE (Muff, Held & Keller, 2016).

### Implications for saving birds

As studies of bird window collisions accumulate, results underscore the point that most windows and vegetation that attracts birds near windows are dangerous. An extensive review shows that 29%, or nearly 3 billion birds, have been lost from North American bird population numbers of 1970, with 57% of species experiencing declines (Rosenberg et al., 2019). In addition to the estimate of up to a billion lost yearly due to window collisions in the US (Loss et al., 2014), there are threats from climate change, habitat loss, domestic cats, and other built structures (Loss, Will & Marra, 2015; Rosenberg et al., 2019). Unless there is rapid and widespread adoption of bird-friendly windows and window mitigation tools, the absolute number of window collision threats to birds will increase.

Rough estimates can demonstrate how dangers posed by windows in the US have increased since the 1970s, when 3 billion more birds lived. The median single family detached house grew from 142 m^2^ (1,525 square feet) in 1973 to 214 m^2^ (2,301 square feet) in 2019 (U.S. Census Bureau, 2020). An industry estimate is that 10–20% of single-family detached housing area is dedicated to windows (National Fenestration Rating Council, 2021). By assuming the midpoint of 15% in both years, the window area of the house grew from 21.3 m^2^ in 1973 to 32.1 m^2^ in 2019. The total numbers of single family detached homes grew from 48 million in 1973 (Eggers, Thackeray & Task, 2007) to about 84 million in 2019 (Neal, Goodman & Young, 2020; U.S. Census Bureau, 2020). Using these figures, window areas in single family homes grew from 1,022,400,00 m ^2^to 2,696,400,000 m^2^, an increase of 1,674,000,000 m^2^. In addition, commercial buildings grew from 5.6 billion m^2^ in 1979 (U.S. Energy Information Administration, 2021b) to 9 billion m^2^ in 2018 (U.S. Energy Information Administration, 2021a), although estimates for window areas are unavailable. Nonetheless, with growth of 3.4 billion m^2^ of commercial space and 1.67 million m^2^ in new glass on homes, bird collision deaths have likely increased since the 1970s. The US population is expected to grow by 82.3 million from 2020 to 2060 (Colby & Ortman, 2014). Thus, trends of larger and more numerous buildings are likely to continue growing, creating greater threats to birds over the next few decades, unless our building practices change.

An important possibility for increasing bird protection lies in legislation and new building standards that require bird-friendly windows (American Bird Conservancy, 2021a), which have been adopted by places such as New York City (New York City Department of Buildings, 2020). To create more citizen support of such policies, it will be important to educate citizens about the magnitude of the threats facing birds and to demonstrate that policy and design standards can reduce the threats. We have noticed how few people are aware of bird collisions on our campus; even a grounds crew member believed there were only one or two fatalities per year. People do not generally look at the edges of buildings where most collisions fall. When we are alerted to a collision on campus by those who have learned of our studies it is usually because the bird landed near an entryway or on a well-used sidewalk. Therefore, educational efforts are needed to highlight the magnitude of the problem and its solutions, such as the program offered by the local Great Salt Lake Audubon chapter (Great Salt Lake Audubon, 2021). In addition, posting bird carcasses on a project page for iNaturalist provides a permanent record of the problem for use in classrooms or with bird advocacy groups, as well as for editorial opinion pieces and legislators (Brown, 2020; Winton, Ocampo-Peñuela & Cagle, 2018). Only by highlighting the urgency of the problem and the efficacy of mitigation or prevention measures can we hope to scale up solutions to meet the magnitude of the problem.

## Conclusions

The current study is the third in a series of studies that establish that two separate phases of installation of Feather Friendly^®^ bird collision deterrent is effective in lowering collisions against both mirrored and transparent glass windows on two different buildings. In addition, the studies have provided correlational evidence that permanent bird-friendly windows—ORNILUX^®^ Mikado UV reflective windows and fritted windows—are associated with fewer collisions and that mirrored glass is associated with more collisions. The special dangers created for Cedar Waxwings by pear trees planted near transparent and mirrored windows underscore how human preferences for aesthetic advantages can create ecological traps for birds drawn to food sources near windows. Although these results could benefit from replications in different seasons and places, the accumulating evidence supports preventive actions. We should install protective windows in new construction, mitigate dangerous existing windows, and provide safe food and shelter opportunities for birds when creating landscapes near buildings.

## Supplemental Information

10.7717/peerj.11867/supp-1 Supplemental Information 1 Raw data and code for evaluating intervention and for the correlational analysis and list of species

## Additional Information and Declarations

### Data Availability

The following information was supplied regarding data availability: The data and statistical syntax files for both the mitigation and the correlational study are available in the Supplemental File.
